# Supplementary material for: From sequence to enzyme mechanism using multi-label machine learning
Source: BMC Bioinformatics. 2014 May 19;15:150. doi: 10.1186/1471-2105-15-150 (PMC4229970; doi:10.1186/1471-2105-15-150)
Supplement: Additional file 2 — Java code of ml2db. Additional file ml2db_code.tar.gz contains the Java source code to run the multi-label machine learning experiments and save the results to database. The code’s Javadoc is included. [file 1471-2105-15-150-S2.zip › additional file 2/ml2db/ml2db/doc/test/ml2db/alltests/AllTests.html]

AllTests


JavaScript is disabled on your browser.


- Overview
- Package
- Class
- Use
- Tree
- Deprecated
- Index
- Help

- Prev Class
- Next Class

- Frames
- No Frames

- All Classes

- Summary:
- Nested |
- Field |
- Constr |
- Method

- Detail:
- Field |
- Constr |
- Method


test.ml2db.alltests

## Class AllTests

- java.lang.Object
- - test.ml2db.alltests.AllTests

- ---

    

  ```
  public class AllTests
  extends java.lang.Object
  ```

  All tests Note: RUN AllDataTests FIRST to generate the database tables, some
  arff files, some data etc. WARNING! RUNNING all tests at once may generate
  problems with your database number of open connections etc. You may consider
  running each block of tests separately. Eg. AllDataTests, then AllArffTests
  etc.

  Version:
  :   18 Feb 2008

  Author:
  :   Luna De Ferrari ldf(at)st-andrews.ac.uk

- - ### Constructor Summary

    Constructors

    Constructor and Description

    `AllTests()`
  - ### Method Summary

    Methods

    | Modifier and Type | Method and Description |
    | --- | --- |
    | `static void` | `main(java.lang.String[] args)` |
    | `static junit.framework.Test` | `suite()` |

    - ### Methods inherited from class java.lang.Object

      `equals, getClass, hashCode, notify, notifyAll, toString, wait, wait, wait`

- - ### Constructor Detail


    - #### AllTests

      ```
      public AllTests()
      ```
  - ### Method Detail


    - #### main

      ```
      public static void main(java.lang.String[] args)
      ```


    - #### suite

      ```
      public static junit.framework.Test suite()
      ```


- Overview
- Package
- Class
- Use
- Tree
- Deprecated
- Index
- Help

- Prev Class
- Next Class

- Frames
- No Frames

- All Classes

- Summary:
- Nested |
- Field |
- Constr |
- Method

- Detail:
- Field |
- Constr |
- Method
